# The complete mitochondrial genome of *Reticulitermes labralis* and implications for Rhinotermitidae taxonomy

**DOI:** 10.1080/23802359.2016.1174085

**Published:** 2016-06-20

**Authors:** Ping Wang, Jinlong Zhu, Min Wang, Yousen Zhang, Jun Wang, Yanyan Zhu, Pingping Zhang

**Affiliations:** a School of Life Sciences, Anhui University, Hefei, Anhui, PR China;; b Hefei Termite Control Institute, Hefei, Anhui, PR China

**Keywords:** Mitochondrial genome, phylogenetic analysis, *Reticulitermes labralis*

## Abstract

The complete nucleotide sequence of the mitochondrial genome of *Reticulitermes labralis* was analyzed. This mitochondrial genome is a circular molecule of 15,914 bp in length and has the same gene content and organization as that found in other *Reticulitermes* species. It contains 13 protein-coding genes (PCGs), 22 tRNAs, two rRNAs (12S and 16S rRNA) and a non-coding AT-rich region (CR). The total base composition is strongly biased toward A + T nucleotides (65.1%). Most of the genes are encoded on the H strand, except for the other four protein-coding genes and eight tRNA genes on the L strand. The phylogenetic tree based on the nucleotide sequences of 13 mitochondrial PCGs using the Bayesian inference method supports the traditional morphologically analysis.

Termites rank as the invasive pests, are well known for their ability to damage wooden structures all over the world (Cameron et al. 2012). The *Reticulitermes labralis* is a species of Rhinotermitidae family which has been widely distributed in north and central China. The mitogenome sequence as a new molecular markers are extremely useful in species-level and population genetic studies (Tokuda et al. 2012). The specimens of *R. labralis* were collected from the Zipeng Mountain in Hefei, Anhui Province, China (31°74′61.37″N, 117°02′78.44″E). The specimens were preserved in 95% ethanol and stored at −20 °C until DNA extraction. The whole genomic DNA was extracted from the heads of 10 soliders of *R. labralis*. The nucleotide sequences of *R. labralis* mitochondrial genome were determined after PCR amplification, sequencing and annotations.

The complete mtDNA of *R. labralis* was a closed circular molecule of 15,914 bp long (accession no. KU877221) and consisted of 13 PCGs, 22 tRNA genes, 2 rRNA genes and a non-coding AT-rich region (CR). Among these, the L strand encoded 14 genes, including eight tRNA genes (*tRNA^Gln^, tRNA^Cys^, tRNA^Tyr^, tRNA^Phe^, tRNA^His^, tRNA^Pro^, tRNA^Leu(CUN)^* and *tRNA^Val^*), four PCGs (*ND5, ND4, ND4L* and *ND1*) and two rRNA genes (12S and 16S rRNA), and the other 23 genes were encoded on the H strand. The total base composition was strongly biased toward A + T nucleotides (65.1%), as commonly characteristic observed in insect mitochondrial genomes (Wei et al. 2010). The 16S rRNA and 12S rRNA genes were located between *tRNA^Leu(CUN)^* and *tRNA^Val^* and between *tRNA^Val^* and CR, respectively. They were 1302 bp and 742 bp long. For R. *labralis,* the order and the orientation of the gene were identical to those previously reported from *Reticulitermes* species (Bonen et al. 2007).

Fourteen related termite species were used to reconstruct phylogenetic tree based on the 13 mitochondrial PCGs through the Bayesian inference (BI) method and *Mastotermes darwiniensis* (GenBank accession no. JX144929) was selected as the outgroup. The Bayesian analysis was performed using the MrBayes version 3.1.2 software (Huelsenbeck & Ronquist 2001). In this process, the best-fitting nucleotide substitution model (GTR + I + T) was selected via Modeltest version 3.06 (Posada & Crandall 1998); the Markov Chain Monte Carlo (MCMC) was run with four chains (one cold chain and three hot chains) for 1,000,000 generations until the average standard deviation of split frequencies reached a value less than 0.01, with Bayesian posterior probabilities calculated from the sample points after the MCMC algorithm had started to converge.

The result of phylogenetic tree among the 14 termite species was shown in Figure 1. It was clearly seen that the phylogenetic tree was divided into two major clades, the first lineage, family Termitidae, included *Macrotermes* and *Odontotermes* genus. The second lineage, family Rhinotermitidae, included *Reticulitermes, Coptotermes* and *Heterotermes.* The conclusion that *Reticulitermes* was a sister group to *Coptotermes and Heterotermes* was consistent with other studies (Ohkuma et al. 2004). The molecular-based phylogeny supported the traditional morphologically analysis. Our study of *R. labralis* could provide a useful database for analyzing the classification and status in Rhinotermitidae.

**Figure 1.**
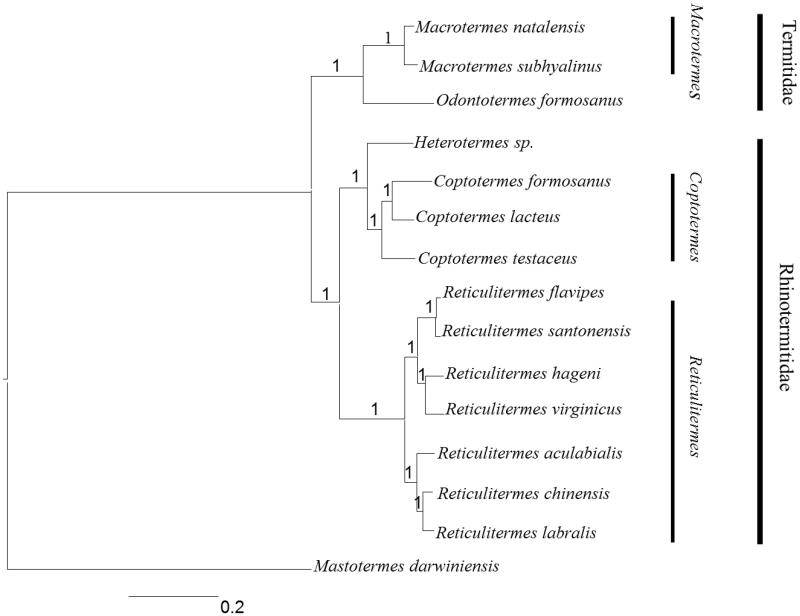
Inferred phylogenetic relationships among termite based on the nucleotide sequences of 13 mitochondrial PCGs using Bayesian inference (BI). Numbers at each node indicate percentages of Bayesian posterior probabilities (BPPs). GenBank accession numbers for the published sequences are KP334994.1 (*Reticulitermes aculabialis*), KM216388.1 (*Reticulitermes chinensis*), EF206314.1(*Reticulitermes flavipes*), EF206320.1 (*Reticulitermes hageni*), EF206315.1 (*Reticulitermes santonensis*), EF206318.1 (*Reticulitermes virginicus*), JX144936.1 (*Heterotermes sp.*), AB626146.1 (*Coptotermes formosanus*), JX144934.1 (*Coptotermes lacteus*), KR872938.1 (*Coptotermes testaceus*), KM405637.1 (*Macrotermes natalensis*), JX144937.1 (*Macrotermes subhyalinus*), KP026254.1 (*Odontotermes formosanus*) and JX144929.1 (*Mastotermes darwiniensis*).
